# Does Left Ventricular Rotational Mechanics Depend on Aortic Valve Annular Dimensions in Healthy Adults?—A Three-Dimensional Speckle-Tracking Echocardiography-Derived Analysis from the MAGYAR-Healthy Study

**DOI:** 10.3390/biomedicines13040817

**Published:** 2025-03-28

**Authors:** Attila Nemes, Nóra Ambrus, Csaba Lengyel

**Affiliations:** Department of Medicine, Albert Szent-Györgyi Medical School, University of Szeged, P.O. Box 427, 6725 Szeged, Hungary; ambrusnora@gmail.com (N.A.); lecs@in1st.szote.u-szeged.hu (C.L.)

**Keywords:** left ventricular, rotation, aortic valve annulus, three-dimensional, speckle-tracking, echocardiography

## Abstract

**Introduction.** There is a balanced relationship between the left ventricle (LV), the aortic valve and the aorta, the functioning of which is essential for optimal circulation. Associations between simultaneously assessed LV rotational mechanics and aortic valve annular (AVA) dimensions respecting the cardiac cycle have never been assessed in clinical circumstances in healthy individuals by three-dimensional speckle-tracking echocardiography (3DSTE). The present study aimed to perform an extensive investigation in order to clarify their possible associations. **Methods.** The present study comprised 111 healthy individuals (mean age 35.3 ± 12.0 years, 69 males). **Results.** With increase in end-diastolic AVA area, tendentious increase in apical LV rotation and consequential LV twist could be detected. Basal and apical rotations and LV twist were tendentiously higher in case of mean end-systolic AVA area compared to lower/higher than mean end-systolic AVA area. With increase in basal LV rotation, tendentious decrease in end-diastolic AVA dimensions could be detected. End-systolic AVA dimensions were tendentiously smaller in case of mean basal LV rotation compared to lower/higher than mean basal LV rotations. With increase in apical LV rotation, tendentious increase in end-diastolic AVA dimensions could be detected. End-systolic AVA dimensions were tendentiously higher in case of mean apical LV rotation compared to lower/higher than mean apical LV rotations. **Conclusions.** No obvious significant association could be detected between simultaneously assessed LV rotational mechanics and AVA dimensions respecting the cardiac cycle in healthy adults.

## 1. Introduction

There is a balanced relationship between the left ventricle (LV), the aortic valve and the aorta, the functioning of which is essential for optimal circulation. This relationship is called ventricular–valvular–vascular coupling [1,2,3]. In this interaction between the LV and the systemic circulation, the aortic valve and its annulus (AVA) play an important role [4]. Modern cardiovascular imaging methods allow a detailed analysis of LV function under physiologic circumstances. LV rotational mechanics have a significant role in optimizing LV ejection [5]; its physiological basis is based on the subendocardial and subepicardial LV muscle fibers running perpendicular to each other [5,6,7,8,9]. Three-dimensional speckle-tracking echocardiography (3DSTE) seems to be an optimal choice for non-invasive easy-to-perform assessment of LV rotational parameters [10,11,12,13]. However, 3D echocardiography has been demonstrated to have a role in the detailed assessment of AVA dimensions as well [14,15]. However, associations between simultaneously assessed LV rotational mechanics and AVA sizes respecting the cardiac cycle have never been evaluated in clinical circumstances in healthy individuals by 3DSTE. Therefore, the present study aimed to perform an extensive investigation in order to clarify their potential associations.

## 2. Materials and Methods

Healthy subjects. The present clinico-physiologic study consisted of 111 healthy adult participants (mean age 35.3 ± 12.0 years, 69 males), who were involved on a voluntary basis between 2011 and 2017. Individuals were healthy based on the fact that all known disorders and other conditions potentially affecting the findings could be excluded in all cases. No obesity, pregnancy or smoking were present, and none of the participants were professional athletes at the time of the enrollment. Physical examination, laboratory, electrocardiography (ECG) and two-dimensional (2D) Doppler echocardiography findings proved to be negative in all cases. Together with routine echocardiography, a 3DSTE-derived data acquisition was also performed. The present retrospective study is a part of the ‘Motion Analysis of the heart and Great vessels bY three-dimensionAl speckle-tRacking echocardiography in Healthy subjects’ (MAGYAR-Healthy) Study, which was partly organized for comparing 3DSTE-derived variables in healthy adults among others (‘Magyar’ means ‘Hungarian’ in Hungarian language). The study was performed in accordance with the Helsinki Declaration (revised in 2013), the Institutional and Regional Biomedical Research Committee of the University of Szeged approved it under the registration number of 71/2011, and informed consent was given by all healthy individuals.

Two-dimensional Doppler echocardiography. All routine 2D Doppler echocardiographic studies were carried out by the same Toshiba Artida^TM^ echocardiography device (Toshiba Medical Systems, Tokyo, Japan), which was attached to a PST-30BT (1–5 MHz) phased-array transducer. The examinations were performed in lateral decubitus position, the patients were asked to lie on his/her left side on the examination table, facing away from the examiner, then the transducer was placed on the typical parasternal and apical positions by the examiner. Chamber quantifications were performed including measurement of LA and LV dimensions with determination of Simpson’s LV ejection fraction (EF) in accordance with the guidelines [16]. Significant valvular regurgitations and stenoses were excluded by Doppler echocardiography, together with the measurement of transmitral flow early and late diastolic E and A velocities and their ratio. 

**Figure 1:**
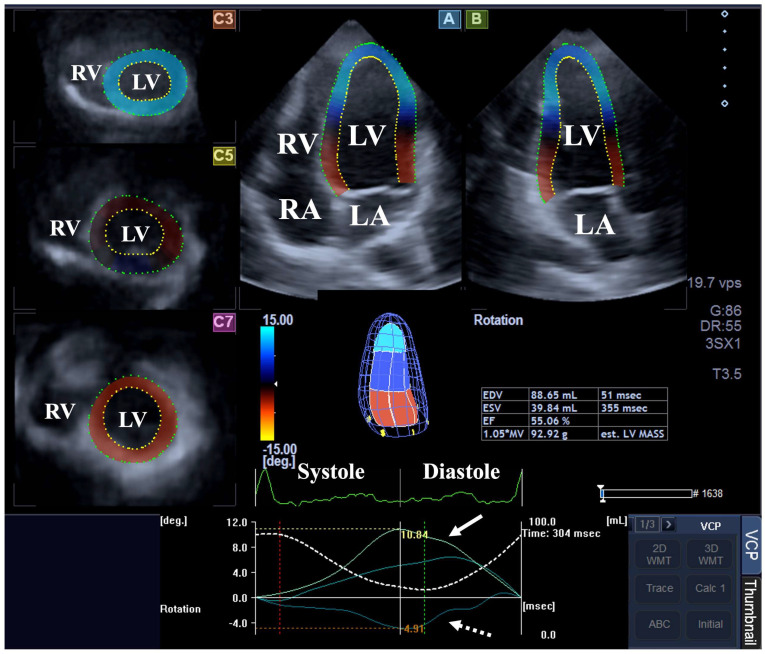
Assessment of the left ventricular (LV) rotational parameters by three-dimensional (3D) speckle-tracking echocardiography. Apical long-axis four-chamber (A) and two-chamber (B) views and short-axis views at the basal (C3), midventricular (C5) and apical LV levels (C7) are shown with a virtual 3D model of the LV and calculated LV volumetric data. Apical (white arrow) and basal (white dashed arrow) time—LV rotational curves are presented together with a curve representing time—LV volume changes (dashed white line) during the heart cycle. Abbreviations: LA = left atrium; LV = left ventricle; RA = right atrium; RV = right ventricle; EDV = end-diastolic volume; ESV = end-systolic volume; EF = ejection fraction.

Three-dimensional speckle-tracking echocardiography. In all cases, 3DSTE has been performed in 2 steps [10,11,12,13]. Firstly, 3D echocardiographic datasets were acquired by the same Toshiba echocardiography machine after changing the transducer to a PST-25SX matrix phased-array transducer. Following image optimizations (gain, magnitude, etc.), the apical window was used for acquisition of 6 subvolumes within 6 cardiac cycles during a single breath hold for optimal images. As a second step, 3D Wall Motion Tracking software (version 2.7, Ultra Extend, Toshiba Medical Systems, Tokyo, Japan) was used for data analysis using auto-created, full volume, pyramid-shaped 3D echocardiographic datasets. Data have been presented in apical long-axis four-chamber (AP4CH) and two-chamber (AP2CH) views and 3 (basal, midventricular and apical) cross-sectional views created automatically by the software. Septal and lateral edges of the mitral annulus - LV and the reference points of the endocardial surface of the LV apex were determined by the examiner, a sequential analysis was started with automatic contour detection, then a virtual 3D LV cast has been created. The following parameters of LV rotational mechanics have been measured: basal and apical LV rotations, LV twist and time-to-LV twist (Figure 1). 

**Figure 2:**
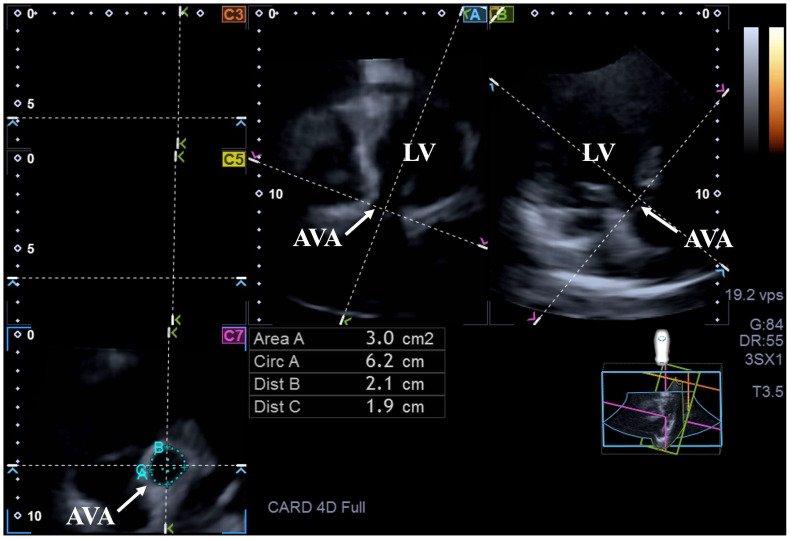
Assessment of the aortic valve annular dimensions by three-dimensional speckle-tracking echocardiography. Abbreviations: LV = left ventricle; AVA = aortic valve annulus, Area = AVA area, Circ = AVA perimeter, Dist B = maximum AVA diameter, Dist C = minimum AVA diameter.

For assessment of AVA dimensions, following optimization of LV longitudinal planes on AP4CH and AP2CH views, the aortic valve and the aorta were visualized by tilting and optimizing the longitudinal planes in AP4CH and AP2CH views; these planes were aligned parallel to the centerline of the aortic root. Then, C7 cross-sectional view perpendicular to the longitudinal plane has been aligned to the AVA. Special attention has to be paid during assessment, on the one hand, to ensure that this C7 plane is perpendicular, and, on the other hand, to make sure that the real AVA is used, not the outflow tract or the Valsalva. With this method, the following features of the AVA have been measured: maximum and minimum AVA diameters (Dmax and Dmin, respectively), areas (A) and perimeters (P) in end-diastole (ED) and end-systole (ES) (Figure 2).

Statistical analysis. All continuous or categorical variables were presented in mean ± standard deviation (SD) and number/percentage formats. In case of *p* < 0.05, statistical significance was considered to be present. Data analyses were carried out by independent sample *t*-test, analysis of variance (ANOVA) or Kruskal-Wallis H tests, where appropriate. Bonferroni correction for multiple comparisons has been performed. For correlations, Pearson correlation coefficients were calculated. To test reproducibility of 3DSTE-derived measurement of LV rotational parameters and AVA dimensions, the mean ± standard deviation difference in values obtained by two measurements of the same observer (intraobserver agreement) and by two observers (interobserver agreement) were tested in 25 healthy individuals together with the respective interclass correlation coefficients (ICCs). For statistical analyses, SPSS software (version 22, SPSS Inc., Chicago, IL, USA) was used.

## 3. Results

Demographic and clinical parameters. Routine parameters including systolic and diastolic blood pressures (121.9 ± 3.0 mmHg and 82.9 ± 1.8 mmHg, respectively), heart rate (70.5 ± 1.4 1/s), height (168.2 ± 9.4 cm) and weight (72.6 ± 14.2 kg) were in the normal reference ranges in all cases. 

2D Doppler echocardiographic and 3DSTE data. All routine 2D echocardiographic parameters proved to be in the normal reference ranges including LA diameter measured in parasternal long-axis view (37.4 ± 3.8 mm) and LV end-diastolic diameter and volume (48.2 ± 3.8 mm and 107.1 ± 23.7 ml, respectively), LV end-systolic diameter and volume (32.1 ± 3.3 mm and 38.1 ± 9.3 ml, respectively), interventricular septum and LV posterior wall (9.3 ± 1.3 mm and 9.5 ± 1.5 mm, respectively), LV-EF (64.8 ± 4.0%) and early and late mitral inflow velocities (78.3 ± 16.6 and 59.2 ± 14.2 cm/s, respectively), all measured in AP4CH and AP2CH views. None of the healthy individuals showed > grade 1 valvular regurgitation or significant valvular stenosis on any valves. Basal and apical LV rotations, LV twist and time-to-LV twist proved to be −4.06 ± 2.29 degrees, 9.33 ± 3.82 degrees, 13.39 ± 4.32 degrees and 341 ± 120 ms, respectively. End-diastolic maximum and minimum AVA diameter, AVA area and AVA perimeter were 2.02 ± 0.31 cm, 1.82 ± 0.30 cm, 3.14 ± 0.86 cm^2^ and 6.30 ± 0.87 cm, respectively. The same parameters in end-systole proved to be 2.05 ± 0.30 cm, 1.87 ± 0.28 cm, 3.35 ± 0.87 cm^2^ and 6.50 ± 0.85 cm, respectively. 

Classification of subjects. Healthy individuals were grouped according to the mean ± SD of end-diastolic and end-systolic AVA area and basal and apical LV rotations based on their lower than mean-SD (2.28 cm^2^, 2.48 cm^2^, −1.77 degrees and 5.51 degrees, respectively) and higher than mean+SD (4 cm^2^, 4.22 cm^2^, −6.35 degrees and 13.15 degrees, respectively) values. 

LV rotations in AVA dimension subgroups. Females had smaller, while males had larger AVA areas. Although no significant differences were found, with increase in end-diastolic AVA area, increase in apical LV rotation and consequential LV twist could be detected. Although basal and apical rotations and LV twist were higher in case of mean end-systolic AVA area as compared to these values measured in case of lower/higher than mean end-systolic AVA area, the difference did not reach the level of significance. End-systolic AVA dimensions were larger than end-diastolic ones mostly in the presence of smaller end-diastolic and larger end-systolic AVA areas. With increase in end-diastolic AVA area, all other end-diastolic and all end-systolic AVA dimensions showed parallel increase. Similar changes were present with end-systolic AVA area (Table 1).

**Figure 3:**
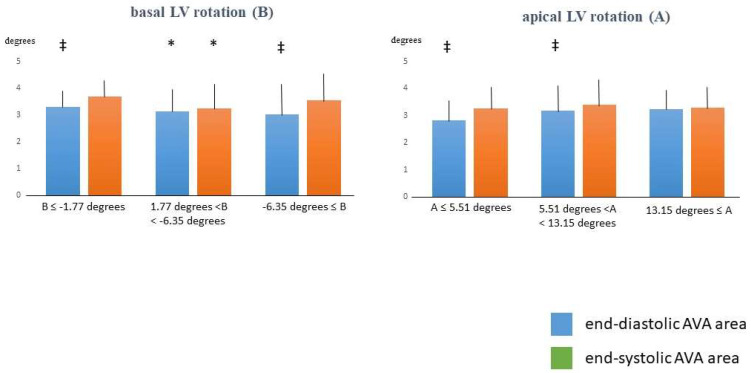
End-diastolic and end-systolic aortic valve annular area in different left ventricular rotations subgroups. * *p* < 0.05 vs. basal LV rotation ≤ −1.77 degrees; ‡ *p* < 0.05 vs. end-systolic counterpart. Abbreviations. B = basal LV rotation, A = apical LV rotation, LV = left ventricular.

AVA dimensions in LV rotation subgroups. Although no significant differences were found in most of cases, with increase in basal LV rotation, decrease in end-diastolic AVA dimensions could be detected. Although end-systolic AVA dimensions were smaller in case of mean basal LV rotation as compared to cases with lower/higher than mean basal LV rotations, the difference did not reach the level of significance. With increase in apical LV rotation, non-significant increase in end-diastolic AVA dimensions could be detected. End-systolic AVA dimensions were non-significantly higher in case of mean apical LV rotation as compared to cases with lower/higher than mean apical LV rotations. Increase in LV basal or apical rotations were associated with increase in LV twist (Table 2, Figure 3). 

Correlation analysis. No correlations could be demonstrated between basal LV rotation and end-diastolic and end-systolic maximum AVA diameter (r = 0.10, *p* = 0.91 and r = 0.03, *p* = 0.78, respectively), minimum AVA diameter (r = 0.12, *p* = 0.22 and r = 0.02, *p* = 0.80, respectively), AVA area (r = 0.08, *p* = 0.42 and r = −0.002, *p* = 0.98, respectively) and AVA perimeter (r = 0.08, *p* = 0.39 and r = 0.001, *p* = 0.99, respectively). Similarly, no correlations were present between apical LV rotation and end-diastolic and end-systolic maximum AVA diameter (r = 0.08, *p* = 0.39 and r = 0.04, *p* = 0.72, respectively), minimum AVA diameter (r = 0.10, *p* = 0.29 and r = 0.007, *p* = 0.94), AVA area (r = 0.11, *p* = 0.26 and r = −0.01, *p* = 0.89) and AVA perimeter (r = 0.12, *p* = 0.21 and r = −0.003, *p* = 0.97).

Intraobserver and interobserver agreements. The mean ± 2 SD difference in values obtained by two measurements of the same observer and by two observers for the assessments of 3DSTE-derived LV rotational parameters and AVA dimensions with respective ICCs are presented in Table 3.

## 4. Discussion

Rotational mechanics of the LV play a fundamental role in optimizing ejection in systole, accounting for up to 40% of it based on physiological studies [5,6,7,8,9]. However, many factors influence the degree of LV rotational mechanics even in healthy circumstances. Not only can the balance between the subendocardium and the subepicardium, the degree of contraction and relaxation and LV myocardial fiber orientation affect LV rotational mechanics [5], but also the volumes and deformations of the LA and LV and the size of MA respecting the cardiac cycle among others [17,18,19,20,21]. Increased apical LV rotation was found to be associated with reduced LV volumes [17], LV strains in all directions [18] and left atrial (LA) thinning [19], but did not show obvious associations with LA volumes [20]. However, basal LV rotation showed specific associations with LV volumes [17], but had an inverse relationship with global LV circumferential strain [18], and associated with LA widening [19] and volumes [20]. While apical LV rotation correlated with end-systolic MA dimensions and functional properties, but not with end-diastolic ones, basal LV rotation did not show any associations with MA parameters [21]. Since the LV is ejected towards the aorta through the aortic valve and AVA, the degree of LV rotational mechanics has been shown to depend on aortic stiffness as well, which can be considered as a special aspect of ventricular-arterial coupling [2,22]. According to these facts, it seems to be reasonable to ask what relationship can be confirmed between the dimensions of the AVA and LV rotational mechanics in healthy adults.

In recent decades, cardiovascular imaging has undergone enormous development, and today there is a wide range of possibilities for examining heart chambers and valves. A new window to the heart has been opened up by 3D echocardiography like 3DSTE, as it is not only capable of volumetric and strain/rotational analysis of the LV simultaneously, but also allows the determination of valvular annuli including AVA respecting to cardiac cycle after setting the optimal planes [10,11,12,13,14,15]. There has been validation of 3DSTE for LV rotational mechanics [23,24,25]. This is the first study using such imaging to examine the relationship between LV rotational mechanics and AVA size in healthy subjects. 

The present investigation suggests a number of implementations. First of all, it has been confirmed that 3DSTE is capable of performing simultaneous analysis of LV rotational parameters and AVA dimensions respecting the cardiac cycle and evaluating their relationship at the same time using the same 3D echocardiographic dataset. Secondly, significant associations could not be detected; only tendencies and non-significant differences were found between these parameters. It seems that basal and apical LV rotations adapt differently and oppositely to the end-diastolic AVA dimensions. Moreover, the different response pattern of LV rotational mechanics to end-diastolic and end-systolic AVA sizes and the different response pattern of end-diastolic and end-systolic AVA sizes to different degree of LV rotational mechanics are suggested to be present, which requires further investigations in larger populations with more detailed methods. Thirdly, it would be interesting to know whether these sorts of associations are present in certain disorders and what significance this has. 

Limitation section. The most important limitations of the study are listed below:

1. Only a small number of healthy subjects have been involved in the present retrospective study in which complete 2D Doppler echocardiography extended with 3DSTE has been performed. Further studies with a larger number of healthy people are warranted following appropriate power analysis to make the whole analysis statistically stronger. The retrospective design of the present study may lead to selection bias, which should be considered when interpreting results as well;
2. Females have smaller, whole males have larger AVA dimensions as demonstrated above, which suggest effects of gender distribution on findings suggesting further investigations in this topic [26];
3. There are significant qualitative differences between the images taken during 2D echocardiography and 3DSTE. The routine 2D echocardiography still comes with significantly better image quality and spatial and temporal resolution, which partially limits the use of 3DSTE in daily clinical practice. The average frame rate achievable during 3DSTE is low (32 ± 3 fps). Another important factor is the size of the transducer, which is for 3DSTE larger than the one used in 2D echocardiography, limiting the efficiency of data acquisitions. Finally, during the processing of the data, digital acquisition of six subvolumes during six cardiac cycles occur, which may increase the chance of creating a stitching or a movement artifact. All these technical problems could have effects on the measured data [10,11,12,13,16,27];
4. Although a 3DSTE offers simultaneous assessment of the number of LV functional features like strains, the present study aimed to assess only LV rotational parameters [28];
5. Moreover, 3DSTE is also capable of creating 3D virtual models of other cardiac chambers, but the present study did not aim for such analyses;
6. 3DSTE-based measurement of parameters featuring LV rotational mechanics are validated; therefore, further validation in the present study was not aimed to be performed again.

## 5. Conclusions

No obvious significant associations between simultaneously assessed LV rotational mechanics and AVA dimensions respecting the cardiac cycle could be detected in healthy adults.

## Figures and Tables

**Table 1:** Aortic valve annular dimensions and left ventricular rotational parameters in different aortic valve annular groups.

	ED-AVA-A ≤2.28 cm^2^ (n = 15)	2.28 cm^2^ < ED-AVA-A < 4 cm^2^ (n = 77)	ED-AVA-A ≥4 cm^2^ (n = 19)	ES-AVA-A ≤2.48 cm^2^ (n = 11)	2.48 cm^2^ < ES-AVA-A <4.22 cm^2^ (n = 81)	ES-AVA-A ≥4.22 cm^2^ (n = 19)
mean age (years)	33.9 ± 12.2	35.7 ± 12.0	37.6 ± 2.5	36.2 ± 11.2	35.5 ± 11.5	33.2 ± 10.7
males (%)	4 (27)	49 (64) *	16 (84) *	1 (9)	40 (49) **	18 (95) **††
LV rotational mechanics						
basal LV rotation (°)	−4.60 ± 2.91	−3.96 ± 2.15	−4.03 ± 2.26	−3.90 ± 1.95	−4.12 ± 2.26	−3.89 ± 2.59
apical LV rotation (°)	7.82 ± 4.03	9.48 ± 3.69	9.93 ± 3.88	8.06 ± 4.11	9.50 ± 3.84	9.38 ± 3.43
LV twist (°)	12.42 ± 5.32	13.44 ± 4.06	13.96 ± 4.34	11.95 ± 4.62	13.61 ± 4.17	13.26 ± 4.60
LV twist time (ms)	384 ± 180	342 ± 104	301 ± 108	380 ± 146	345 ± 119	300 ± 96
Aortic valve annulus						
ED-AVA-Dmax (mm)	1.56 ± 0.17 ‡	2.01 ± 0.20 *	2.44 ± 0.23 *†	1.65 ± 0.25	1.99 ± 0.25 **	2.36 ± 0.29 **††‡
ED-AVA-Dmin (mm)	1.35 ± 0.10 ‡	1.82 ± 0.20 *	2.19 ± 0.16 *†	1.41 ± 0.15	1.79 ± 0.24 **	2.15 ± 0.22 **††
ED-AVA-A (mm)	1.80 ± 0.27 ‡	3.07 ± 0.44 *‡	4.46 ± 0.51 *†	1.98 ± 0.46	3.06 ± 0.64 **‡	4.14 ± 0.79 **††‡
ED-AVA-P (mm)	4.84 ± 0.41 ‡	6.27 ± 0.45 *‡	7.58 ± 0.40 *†	5.02 ± 0.59	6.25 ± 0.66 **‡	7.26 ± 0.70 **††‡
ES-AVA-Dmax (mm)	1.72 ± 0.19	2.04 ± 0.24 *	2.37 ± 0.27 *†	1.61 ± 0.18	2.02 ± 0.20 **	2.48 ± 0.19 **††
ES-AVA-Dmin (mm)	1.57 ± 0.16	1.85 ± 0.24 *	2.17 ± 0.21 *†	1.43 ± 0.17	1.84 ± 0.20 **	2.23 ± 0.18 **††
ES-AVA-A (mm)	2.35 ± 0.40	3.28 ± 0.63 *	4.43 ± 0.82 *†	1.92 ± 0.28	3.23 ± 0.47 **	4.73 ± 0.50 **††
ES-AVA-P (mm)	5.45 ± 0.50	6.46 ± 0.62 *	7.54 ± 0.71 *†	4.99 ± 0.40	6.42 ± 0.49 **	7.75 ± 0.44 **††

* *p* < 0.05 vs. ED-AVA-A ≤ 2.28 cm^2^; † *p* < 0.05 vs. 2.28 cm^2^ < ED-AVA-A < 4 cm^2^; ** *p* < 0.05 vs. ES-AVA-A ≤ 2.48 cm^2^; †† *p* < 0.05 vs. 2.48 cm^2^ < ES-AVA-A < 4 cm^2^; ‡ *p* < 0.05 vs. end-systolic counterpart. **Abbreviations.** ES = end-systolic, ED = end-diastolic, AVA = aortic valve annulus, Dmax = maximum AVA diameter, Dmin = minimum AVA diameter, A = AVA area, P = AVA perimeter.

**Table 2:** Aortic valve annular dimensions and left ventricular rotational parameters in different left ventricular rotational parameter subgroups.

	Basal LV Rotation ≤ −1.77 Degrees (n = 14)	−1.77 Degrees < Basal LV Rotation < −6.35 Degrees (n = 77)	Basal LV Rotation ≥ −6.35 Degrees (n = 20)	Apical LV Rotation ≤ 5.51 Degrees (n = 16)	5.51 Degrees < Apical LV Rotation < 13.15 Degrees (n = 77)	Apical LV Rotation ≥ 13.15 Degrees (n = 18)
mean age (years)	36.1 ± 11.7	34.0 ± 11.1	39.6 ± 14.4	36.8 ± 12.5	34.7 ± 12.0	36.7 ± 11.6
males (%)	11 (79)	44 (57)	14 (70)	10 (63)	46 (60)	13 (72)
LV rotational mechanics						
basal LV rotation (°)	−1.14 ± 0.60	−3.55 ± 1.09 *	−8.06 ± 1.18 *†	−4.03 ± 2.76	−4.11 ± 2.30	−3.86 ± 1.75
apical LV rotation (°)	9.57 ± 3.48	9.59 ± 3.92	8.67 ± 3.39	3.18 ± 1.64	9.20 ± 1.91 **	15.36 ± 1.38 **††
LV twist (°)	10.70 ± 3.70	13.12 ± 3.92	16.72 ± 3.70 *†	7.20 ± 3.40	13.31 ± 2.69 **	19.23 ± 2.40 **††
LV twist time (ms)	289 ± 127	349 ± 124	343 ± 88	311 ± 173	340 ± 106	353 ± 120
Aortic valve annulus						
ED-AVA-Dmax (mm)	2.08 ± 0.22	2.02 ± 0.32	1.98 ± 0.34 ‡	1.96 ± 0.37	2.02 ± 0.31 ‡	2.09 ± 0.25
ED-AVA-Dmin (mm)	1.95 ± 0.16	1.81 ± 0.30 *	1.78 ± 0.32 ‡	1.71 ± 0.31	1.84 ± 0.30 ‡	1.83 ± 0.26
ED-AVA-A (mm)	3.30 ± 0.53 ‡	3.14 ± 0.84 *	3.02 ± 1.05 ‡	2.83 ± 0.78 ‡	3.18 ± 0.89 ‡	3.24 ± 0.69
ED-AVA-P (mm)	6.49 ± 0.52 ‡	6.31 ± 0.87 *	6.16 ± 1.03 ‡	6.00 ± 0.90 ‡	6.33 ± 0.90 ‡	6.44 ± 0.67
ES-AVA-Dmax (mm)	2.19 ± 0.23	2.02 ± 0.29 *	2.10 ± 0.35	1.97 ± 0.29	2.07 ± 0.31	2.06 ± 0.25
ES-AVA-Dmin (mm)	2.01 ± 0.17	1.83 ± 0.29 *	1.92 ± 0.29	1.80 ± 0.30	1.90 ± 0.27	1.81 ± 0.29
ES-AVA-A (mm)	3.69 ± 0.65	3.25 ± 0.83 *	3.54 ± 1.03	3.26 ± 0.77	3.39 ± 0.91	3.28 ± 0.74
ES-AVA-P (mm)	6.86 ± 0.61	6.40 ± 0.84 *	6.65 ± 0.94	6.37 ± 0.80	6.55 ± 0.89	6.43 ± 0.66

* *p* < 0.05 vs. basal LV rotation ≤ −1.77 degrees; † *p* < 0.05 vs. −1.77 degrees < basal LV rotation < −6.35; ** *p* < 0.05 vs. apical LV rotation ≤ 5.51 degrees; †† *p* < 0.05 vs. 5.51 degrees < apical LV rotation < 13.15 degrees; ‡ *p* < 0.05 vs. end-systolic counterpart. Abbreviations. ES = end-systolic, ED = end-diastolic, AVA = aortic valve annulus, Dmax = maximum AVA diameter, Dmin = minimum AVA diameter, A = AVA area, P = AVA perimeter.

**Table 3:** Intraobserver and interobserver agreement in measurement of three-dimensional speckle-tracking echocardiography-derived left ventricular rotational parameters and aortic valve annular dimensions.

	Intraobserver Agreement		Interobserver Agreement	
	mean ± 2SD Difference in Values Obtained by 2 Measurements of the Same Observer	ICC Between Measurements of the Same Observer	mean ± 2SD Difference in Values Obtained by 2 Observers	ICC Between Independent Measurements of 2 Observers
basal LV rotation (°)	0.3 ± 0.1	0.81 (*p* < 0.01)	0.3 ± 0.2	0.80 (*p* < 0.01)
apical LV rotation (°)	0.05 ± 0.05	0.80 (*p* < 0.01)	0.6 ± 0.7	0.81 (*p* < 0.01)
ED-AVA-Dmax (mm)	−0.06 ± 0.19	0.85 (*p* < 0.01)	−0.04 ± 0.20	0.89 (*p* < 0.01)
ED-AVA-Dmin (mm)	−0.02 ± 0.24	0.91 (*p* < 0.01)	−0.03 ± 0.20	0.93 (*p* < 0.01)
ED-AVA-A (mm)	−0.14 ± 0.62	0.95 (*p* < 0.01)	−0.12 ± 0.57	0.96 (*p* < 0.01)
ED-AVA-*p* (mm)	−0.05 ± 0.70	0.91 (*p* < 0.01)	−0.13 ± 0.66	0.92 (*p* < 0.01)
ES-AVA-Dmax (mm)	0.01 ± 0.29	0.92 (*p* < 0.01)	0.03 ± 0.31	0.93 (*p* < 0.01)
ES-AVA-Dmin (mm)	0.05 ± 0.33	0.82 (*p* < 0.01)	0.05 ± 0.32	0.82 (*p* < 0.01)
ES-AVA-A (mm)	0.15 ± 0.73	0.92 (*p* < 0.01)	0.12 ± 0.74	0.94 (*p* < 0.01)
ES-AVA-P (mm)	−0.02 ± 0.54	0.92 (*p* < 0.01)	0.01 ± 0.58	0.93 (*p* < 0.01)

Abbreviations. AVA = aortic valve annulus, LV = left ventricular, ED = end-diastolic, ES = end-systolic, Dmax = maximum AVA diameter, Dmin = minimum AVA diameter, A = AVA area, P = AVA perimeter.

## Data Availability

The data presented in this study are available on request from the corresponding author. The data are not publicly available due to [local restrictions].
